# Apocrine Hidrocystoma Mimicking Breast Lesion in a Child from Tunisia

**Published:** 2020-02

**Authors:** Mahdi Ben DHAOU, Mohamed ZOUARI, Salma AMMAR, Naourez GOUIAA, Manel HAJ MANSOUR, Mohamed JALLOULI, Thouraya BOUDAWARA, Riadh MHIRI

**Affiliations:** 1. Department of Pediatric Surgery, Hedi-Chaker Hospital, Sfax, Tunisia; 2. Sfax Medical School, Sfax, Tunisia; 3. Department of Pathology, Habib Bourguiba Hospital, Sfax, Tunisia

## Dear Editor-in-Chief

Hydrocystomas are cystic proliferations of the sweat glands with apocrine or eccrine differentiation (1). Apocrine hidrocystomas arise from the proliferation of apocrine glands and are generally less than 1 cm in diameter (2). The pathogenesis of these tumors is not entirely known, but they tend to appear during adulthood, grow slowly, and persist indefinitely (3, 4). Apocrine hidrocystomas are commonly found on the head, eyelids and neck (5). They are also been reported at other sites, including axillae, areola of the nipple, periumbilical, anal, and genital areas (6). We present a rare case of apocrine hidrocystoma located at the breast area of a child.

Written informed consent was obtained from the legal guardian of the patient to publish this case and accompanying images in scientific journals for research and educational purposes.

**Fig. 1:**
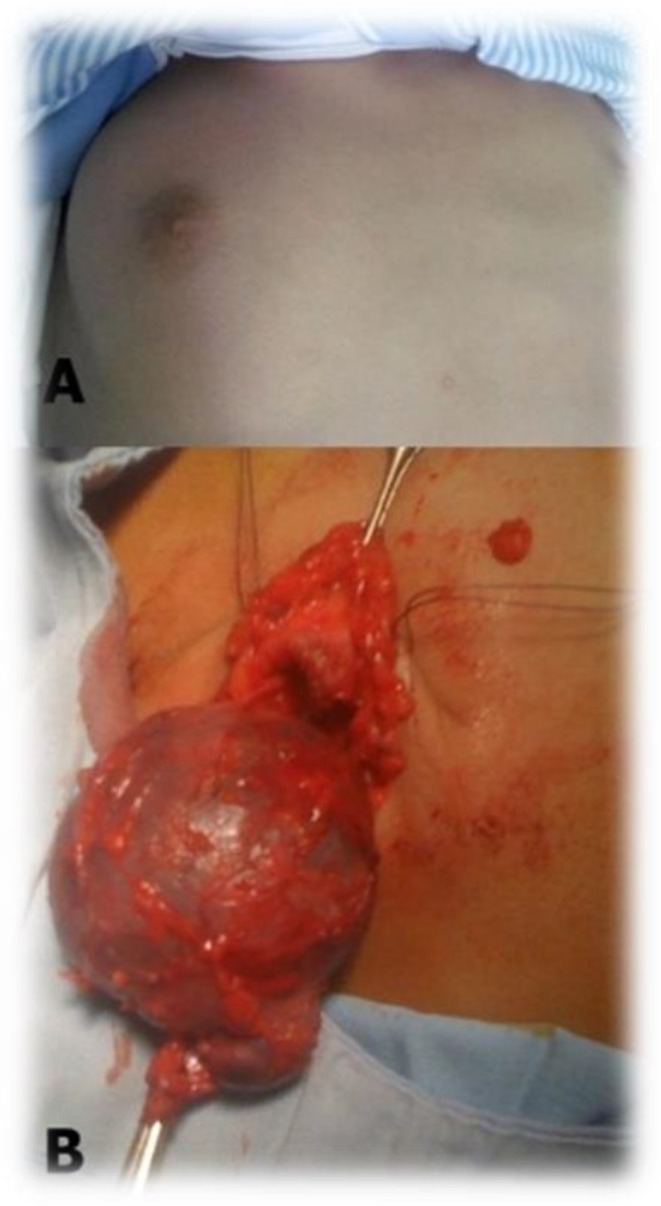
A: Physical examination revealed a cystic mass on the right breast. B: Intraoperative view after the excision of the mass

In January 2015, a healthy 6-yr-old boy presented to the Pediatric Surgery Department (Hedi Chaker Hospital, Sfax, Tunisia) with a painless, cystic mass on the right breast for 2 years. The mass had recently been increasing in size until it was 7×6 cm at presentation (Fig. 1A).

**Fig. 2:**
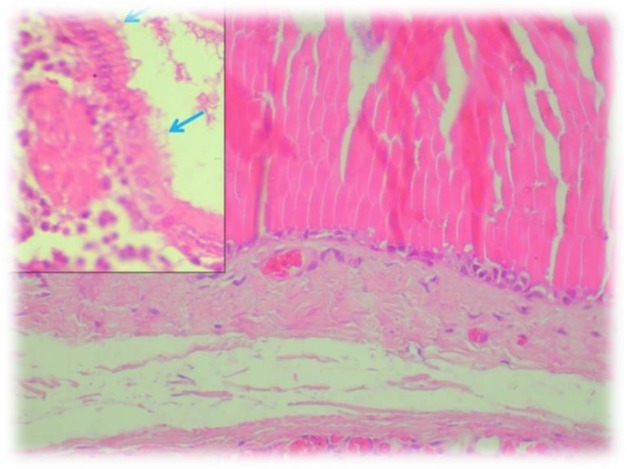
Apocrine hidrocytoma consisting of large multilocular cystic spaces situated in dermis and lined by double layer of cells

He had no past medical history and was otherwise asymptomatic. Ultrasound examination of the right breast mass revealed a 6.5×5 cm hypoechoeic lesion. Differential diagnoses were tending towards cyst lymphangioma rather than a hidrocystoma, clinical suspicion of malignancy was low. The patient proceeded to excision of the lesion and the gross specimen showed characteristics of a multiloculated cyst (Fig. 1B). Histopathology of the tumour revealed a cystic cavity lined by double layer of cells: outer flattened myoepithelial cells and inner columnar cells that display prominent decapitation secretion (Fig. 2); there were no features suggestive of malignancy. These findings were consistent with an apocrine hidrocystoma. The child was free of recurrence at 2 years postoperatively.

Apocrine hidrocystoma is a benign neoplastic lesion of the skin. This tumor is very rare in childhood and generally present as small cutaneous nodules, which are mainly solitary (1). The differential diagnosis of apocrine hidrocystoma includes melanoma, cystic basal cell epithelioma, milium, and epidermoid or pilar cysts, and eccrine hidrocystoma (7, 8). “Histologically, they are unilocular or multilocular dermal cysts lined with cuboidal or high-columnar apocrine secretory cells with decapitation secretion, resting on a layer of elongated myoepithelial cells” (3, 6).

Treatment of single apocrine hidrocystomas should be focussed on excision with narrow margins due to the benign nature of the lesion (6, 9). No further intervention is needed if histological diagnosis is made without question of malignancy. However, knowledge of this histologic diagnosis and understanding is necessary for the management of more serious lesions.
